# Evaluation of The Effects of Psychotherapy on Anxiety Among Mothers of Children With Leukemia

**Published:** 2014

**Authors:** Shiva NAZARI, Nahid MORADI, Mohammad Taghi SADEGHI KOUPAEI

**Affiliations:** 1 Pediatric Congenital Hematologic Disorder Research Center, Shahid Beheshti University of Medical Sciences, Tehran, Iran; 2 Department of Psychology, Islamic Azad University, Roudehen branch, Roudehen, Iran.; 3 Faculty of Paramedical Sciences, Shahid Beheshti University of Medical Sciences, Tehran, Iran

## Abstract

**Objective:**

Children with leukemia and their families face a long period of medical treatment and uncertainty about the future. These families may suffer from short- and long-term emotional problems. The aim of the present study was to assess the effect of supportive psychotherapy on the anxiety of mothers whose children suffer from leukemia.

**Materials & Methods:**

The current research were performed on mothers who had a child with leukemia hospitalized in Mofid Children’s Hospital, Tehran, Iran. They were randomly selected. The research method was a quasi-experimental study with pretest/posttest design. The pretest Kettles’ anxiety questionnaire was given to all the mothers and after seven sessions of supportive psychotherapy, the posttest was performed and the grades were compared.

**Results:**

Ten mothers finished all seven therapeutic sessions. There was a statistically significant difference between the pretest and posttest mean scores, confirming the mothers’ reduced anxiety level.

**Conclusion:**

Finding effective and newer approaches to improve the well-being of parents with a sick child is an important challenge of today’s medical researches. Based on our findings, it is possible to reduce the anxiety in mothers of children with leukemia through supportive psychiatric therapies.

**Keywords: **Leukemia; Mothers; Anxiety; Psychology; Child

## Introduction

Leukemia is the most common malignancy among children, which includes about 30% of all childhood malignancies, and four out of each 100,000 children under the age of 15 become leukemic (1). Although Iran has a lower prevalence of leukemia among children compared to western countries, acute lymphocytic leukemia (ALL) is the most common malignancy in Iranian children (2). 

Studies have demonstrated that the presence of a sick or disabled child in a family can cause stress and depression in parents. This problem is particularly apparent among mothers, since they have a more active role in taking care of the sick child and therefore are in a more stressful position compared to fathers (3,4). Anxiety among parents of leukemic children is a common finding. A study has reported that 51% of mothers and 40% of fathers in first two weeks after their child is diagnosed with cancer develop acute stress based on Diagnostic and Statistical Manual of Mental Disorders DSM-IV (DSM-IV). Another study have found that moderate to severe post-traumatic stress syndrome develops in 68% of mothers and 57% of fathers after their child is diagnosed with leukemia (5,6). This is when taking care of their sick child is a natural and essential task for parents of these patients and the stress and anxiety might reduce the effectiveness of parents in performing this task. In most cases, parents of children with a chronic or severe disease feel guilty, particularly if the disease has a genetic component. These parents sometimes ignore their duties in taking care of their sick child and sometimes develop mental disorders due to feeling guilt. Based on these findings, there is a need for formulation of health and social policies for early diagnosis, proper management of depression and psychosocial problems, and providing required help for families of children with leukemia. Psychiatric treatments are among the methods for effective reduction of mental health problems in parents of patients. Several studies have evaluated the effectiveness of these treatment methods. Supportive psychotherapy establishes a relationship based on feeling of safety and security between therapist and patient and enables the patient to talk about emotions, such as fear, anxiety, sadness, frustration, and irritability (7). In the present study, a combination of techniques for empathic communication, active listening as well as unconditional acceptance of positive thoughts were used, and the effectiveness of these methods on mothers’ anxiety was assessed based on Rogers’ theory. This method of psychotherapy is useful to help the patient during the crisis caused by social problems or chronic psychological and physical illnesses. The aim of the study was to find if supportive psychotherapy based on person-centered Rogers’ theory is effective in reducing the anxiety among mothers of children with leukemia.

## Materials & Methods

The present study was an experimental study with a single group pretest/posttest design. To do the study, first a pretest (using Kettles’ anxiety questionnaire) was performed by a psychological expert, then 7 sessions of supportive psychotherapy were given and then a posttest (using Kettles’ anxiety questionnaire) was taken, and the scores of the pretest and posttest were compared. From the parents of 45 children with leukemia hospitalized in the Mofid Children’s Hospital, Tehran, Iran, mothers of 15 patients were randomly selected to participate in the study. After filling out the Kettles’ anxiety questionnaire by the participants (7), sessions of supportive psychotherapy was performed, with each session lasting 60 minutes. After the final session was performed, the questionnaire was filled out by participants, and the pretest scores were compared with the posttest scores. We followed all moral obligations in conducting this research and no cost was imposed on mothers for entering this study.

Kettles’ anxiety questionnaire is consisted of 40 questions, each of them has three possible answers.

The scoring was performed based on key of the questionnaire. In total, there are three different scores calculated from the results of the questionnaire. 

A. The total score which is calculated from all 40 questions;

B. The score calculated from the first 20 questions showing the undercover anxiety and the score calculated from the last 20 questions, which show the participants’ apparent anxiety;

C. Five scores related to different aspects of participants’ personality are calculated using scores of several indicated questions in the questionnaire;

The Kettles’ anxiety questionnaire has been previously validated for use among Iranian patients (7).

The treatment sessions are summarized as below: 

First session:

The study and its goals were introduced. The goals of the study were: anxiety reduction, raising the levels of faith and spiritual beliefs to boost the moral, giving an opportunity for talking about the problems that were introduced. Also, the importance of the study was discussed. The mothers were asked to introduce themselves (8). This started by filling out the demographic questionnaire, and the Kettles’ questionnaire was answered by the participants.

Second session:

In this session, the caregiver’s goal was to achieve assurance and unconditional acceptance by the mothers. Since the main problem of mothers was the condition of their child, providing a safe and friendly environment, and obtaining the mothers’ assurance by the caregiver would result in mothers feeling safe to talk about their problems. In this session, the caregiver tried to give the mothers an opportunity to feel accepted and understood. Also, the caregiver tried to show a positive and adaptive attitude. This was done by accepting the mothers, the way they are, and valuing them as an individual without considering their appearance or behavior to raise their self esteem.

Third session:

The third session dedicated to actively listening to mothers’ problems and showing empathy. In this session, mothers were encouraged to freely talk about everyday problems. Mothers talked about themselves using verbal and nonverbal communication and also talked about their expectations of their children’s treatment. Empathy was another point in this session, since the mothers would show their feelings by crying. To achieve a relation based on acceptance and assurance, the caregiver tried to understand the world of these mothers and see the world from their perspective. The more caregiver showed feelings and was successful active listening, the more the mothers would freely discuss their feelings, although these feelings were mixed with fear and panic. The caregiver tried to make the therapeutic relation as transparent as possible to give the mothers the opportunity to accept them and feel warmth, compassion, and understanding. 

Forth session:

In this session, after a brief review of previous sessions, the aims of supportive psychotherapy were discussed with the mothers. It was told that the society has long been aware of the needs of its members suffering from physical and psychological problems and has considered helping them to cope with their condition. 

Also, the role of religious organizations in these conditions was discussed.

The goals of supportive psychotherapy, including boosting the self confidence of the patient, informing the patient about realities, and promotion of optimal psychological and social functioning by reinforcing patient’s abilities to follow the life were introduced. 

Also, techniques used to cope with mothers problems, including assurance, unburdening by release of feelings through freely and self concisely talking about those feelings, enlightenment meaning the mothers can understand their emotions and behavior and have a clearer image of reality, were discussed.

Fifth session:

The fifth session was centered on the subject of anxiety, its different kinds, the way they are built, and methods of coping with anxiety. Information regarding the nature of anxiety, including the signs, physical changes, disturbing thoughts, roots of anxiety, social and functional changes caused by it, the difference between anxiety and madness, and the fact that anxiety states are not completely removable from the life but are controllable, were discussed. At the end of this session, mothers were asked to describe their own anxiety conditions. 

Sixth session:

Due to importance of anxiety, this session was also dedicated to further talk about it and its influence on interpersonal relations. Person-centered treatment methods have a positive attitude towards human beings. 

According to Rogers’ theory when patients are put in a position to receive unconditional positive attention, they can freely talk about whatever they like. In our case, this talk was more centered on the mothers’ fears and worries about losing their child. The main feature of this session was release of concealed feelings.

Mothers were free to cry and were happy to have the opportunity to show their emotional reaction, without fear of being judged. Most of the mothers in this session would discuss their personal and family problems due to the warm therapeutic relation established. 

Seventh session:

This session was a rapping up of the treatment process by understanding the patient, empathy, complete acceptance of the patient with unconditional positive attention toward her, showing purity and consistency, and also receiving suggestions. In this session, the caregiver also tried to understand how the mothers think about their condition, what feelings they have, and how they perceive their behavior. To achieve this understanding, there was a need to conveniently live with these mothers, even for a short period of time. Mothers’ judgment about their surrounding world was accepted, not because it was accurate but because it was their judgment and the session was concluded by some suggestions, including the continuation of treatment sessions by some mothers. To evaluate the hypothesis of the study, we used descriptive statistics (mean, standard deviation, and descriptive tables) as well as analytical statistics like t-test. P-values less that 0.05 were considered statistically significant.

## Results

Our study population consisted of 15 randomly selected mothers whose children were hospitalized in our hospital due to leukemia. The scores obtained by mothers in pretest and posttest are presented in Table 1. Considering the fact that only 10 mothers continued the study regularly and to the last session, we are presenting the scores of these 10 subjects. The mean anxiety score for the pretest was 47.10 and the median score was 45. The mean posttest score was 39.30 and the median score was 38. Table 2 shows the standard deviation for these scores. There was a statistically significant difference between the mean anxiety scores before and after treatment sessions (p<0.001) showing a significant reduction of anxiety among the participants.

**Table1:** The Pretest and Posttest Scores’ Mean, Standard Deviation,Median, and Variance

**After the sessions**	**Before the sessions**	
10	10	Number of participants
39.30	47.10	Mean
38.00	45.00	Median
58.32	7.752	Standard deviation
34.011	060.100	Variance

**Table 2:** Correlation Statistics Before and After the Sessions

**Deviation of mean**	**Standard deviation**	**Number of participants**	**Mean**	
2.452	7.752	10	47.10	Before the sessions
1.844	5.832	10	39.30	After the sessions

**Table3:** The Difference Between The Mean Scores Before and After The Sessions

**p-value**	**Degree of freedom**	**T**	**Difference**					
			**Confidence interval**		**Deviation of mean**	**Standard deviation**	**Mean**	
			**Most**	**Least**				
0.001	9	5.016	11.317	4.283	1.555	4.917	7.800	**Before and after the sessions**

## Discussion

The present study showed that supportive psychotherapy based on Rogers’ theory is effective in reducing the anxiety among mothers of patients with leukemia.This is the firststudy performed on the effect of supportive therapy in reducing anxiety among Iranian mothers with a leukemic child. Our findingsare similar to those by Sahler et al. in 2005 (9). In their study, after therapeutic interventions the degree of depression and posttraumatic stress disorder was reduced in mothers of children with newly diagnosed cancer(10). Having a child with cancer causes adverse effects on the psychological well-being of the patients and their family members. The need for medical care and fear of premature death can cause reactions like anger, frustration, hopelessness, and stress among patients and their family. Due to the fact that family is a semi-closed environment, its members should have mutual understanding, and a disease like cancer not only affects the life of the patient, but also has a profound effect on the life of parents and the patient’s siblings, and disrupts family relations. Based on our findings, the level of anxiety before the supportive therapy was high among mothers with a leukemic child, which was expected. Wijberg et al. have reported a similar high anxiety level among parents of children with cancer (11). Another study suggested the same finding by reviewing 67 other studies (12). Yaung et al. in their study of mothers with cancerous children have found that these mothers have difficulty in playing their role in the family, particularly in dealing with their other children (13). In the present study, we tried to reduce the conflict problems among mothers due to their worry about their ill child, since these conflicts could themselves adversely affect their life and their ability in taking care of their leukemic child. Low awareness is another problem among these mothers. In considering this topic, Fisher proposed that diagnosis of a chronic disease in a child causes an emotional crisis in parents (14). In many studies, it has been found that finding the correct information about their child’s disease is one of the most important needs of parents (14). This subject has even been considered in patients with other disease. For example, Stewart et al. in a study on partners of patients with heart attack concluded that improving their information about the disease causes a better compatibility and improves their decision making ability (15). Increasing the mothers’ awareness was achieved in this study. In our country, the subject of emotional status of parents with cancerous child is regularly ignored, but by considering these subject and cost allocation to help these parents, we can help the health of the family and even indirectly help the patient, since parents with a stable mental status can better take care of their cancerous child. Regarding this subject, Kyngas et al. reduction of anxiety among patients and is a crucial predictor of good compliance with therapy (16). 

**In conclusion,** finding effective and newer approaches for improving the well-being of parents with a sick child is an important challenge of today’s medical research. Based on our findings, it is possible to reduce the anxiety among mothers of children with leukemia using supportive psychiatric therapies.
